# Clinical and Economic Implications of Hydroxyurea Intolerance in Polycythemia Vera in Routine Clinical Practice

**DOI:** 10.3390/jcm13123390

**Published:** 2024-06-10

**Authors:** Martin H. Ellis, Tamar Tadmor, Naama Yekutiel, Gabriel Chodick, Moti Levy, Giora Sharf, Nana Ben Zvi, Raanan Leef, Oren Feine, Oren Shavit

**Affiliations:** 1 Hematology Institute, Meir Medical Center, Kfar Saba 4428164, Israel; martinel@clalit.org.il; 2 Faculty of Medicine and Health Sciences, Tel Aviv University, Tel Aviv 6997801, Israel; gabrielchodick@gmail.com; 3 Hematology Unit, Bnai-Zion Medical Center, Haifa 3339419, Israel; tamar.tadmor@b-zion.org.il; 4 The Ruth and Bruce Rappaport Faculty of Medicine, Technion, Haifa 3109601, Israel; 5 Maccabitech Institute of Research and Innovation, Maccabi Healthcare Services, Tel Aviv 6800003, Israel; fund_n@mac.org.il; 6 Flute of Light Patient Advocacy Group, Netanya 4265952, Israel; imotilevy@gmail.com (M.L.); gioras@partner.net.il (G.S.); 7 Novartis Israel Ltd., Tel Aviv 6744129, Israel; nana.ben_zvi@novartis.com (N.B.Z.); oren.feine@gmail.com (O.F.); oren.shavit@novartis.com (O.S.)

**Keywords:** polycythemia vera, hydroxyurea, intolerance, thrombosis, progression, hospitalization, mortality

## Abstract

**Background/Objectives:** Polycythemia vera (PV) is a chronic hematologic neoplasm commonly treated with hydroxyurea (HU). We utilized the advanced digitalized database of Maccabi Healthcare Services to retrospectively investigate the clinical and economic implications of HU intolerance in the routine clinical care of PV patients in Israel. **Methods:** We collected data on demographics, physician visits, hospitalizations, laboratory results, medication purchases, cardiovascular and thrombotic events, mental health, economic outcomes, and mortality. Outcomes included cardiovascular and other thrombotic events, disease progression, mental health events, economic outcomes, and overall mortality. **Results:** Of the 830 patients studied, 3 (0.4%) were resistant to HU treatment, 318 (38.3%) were intolerant to HU treatment, and 509 (61.3%) were stable on HU treatment. The venous thrombosis rate was significantly higher among HU-intolerant compared to HU-stable patients (1.58 vs. 0.47 per 100 person-years [PY], respectively; *p* < 0.001). The rate of progression to myelofibrosis was 6 vs. 0.9 per 100 PY in HU-intolerant patients vs. HU-stable patients, respectively (*p* < 0.001), and the rate of progression to acute myeloid leukemia (AML) was 1.16 vs. 0.2 per 100 PY in HU-intolerant patients vs. HU-stable patients, respectively (*p* < 0.001). The phlebotomy requirement, mortality rate, and total hospitalization days among HU-intolerant patients were significantly higher than in HU-stable patients (*p* = 0.049, *p* < 0.001, *p* < 0.001, respectively). More mental health-related events were noted in HU-intolerant patients vs. HU-stable patients (*p* = 0.007), and the total healthcare cost ratio was 2.65 for the HU-intolerant patients compared with HU-stable patients. **Conclusions:** This study suggests that HU-intolerant patients are more likely to have worse outcomes than HU-stable patients, highlighting the need for the close monitoring of these patients for disease-related complications or progression.

## 1. Introduction

Polycythemia vera (PV) is the most common myeloproliferative neoplasm (MPN) disease [1,2]. PV prevalence is 22–30 per 100,000 individuals [3]^,^ and its incidence increases with age. Disease manifestations include elevated blood cell counts, a predisposition to thrombosis and hemorrhage, symptoms of hyperviscosity, constitutional symptoms, and, in a proportion of patients, progression to myelofibrosis (MF) and/or transformation to acute myeloid leukemia (AML) [4].

Treatment is based on the maintenance of a hematocrit of <45%, and the administration of low-dose aspirin. In patients with a low risk of thrombosis (age < 60 years and no previous thrombosis), the hematocrit is controlled by phlebotomy, with cytoreductive treatment added in patients resistant or intolerant to phlebotomy. In patients at high risk of thrombosis (age > 60 years and/or previous thrombosis), cytoreductive treatment is indicated, with hydroxyurea (HU) being the most frequently used agent for this purpose [2,5,6]. 

Between 11% and 24% of PV patients receiving HU may develop clinically relevant side effects or resistance [5,7,8,9]. In these patients, second-line treatments include interferon-alpha (IFN-α), ruxolitinib, or busulfan. These treatments, while effective, have side effects and are expensive [10,11,12]. 

Even though Israel has a well-established digital medical records system, providing a unique opportunity to study the epidemiology and treatment outcomes of PV patients, there were no available data pertaining to the epidemiology of PV, treatment paradigm, or outcomes.

While previous studies have shown that HU resistance or intolerance may be associated with disease progression and adverse outcomes [7], the implications of stopping HU because of intolerance are not fully characterized. Filling this knowledge gap may be valuable for clinical and health policy considerations. In this study, we report the clinical and economic implications of HU intolerance in adult PV patients in routine clinical practice.

## 2. Methods

### 2.1. Study Design

This retrospective study used the central database of Maccabi Healthcare Services (MHS). MHS is a nationwide health plan (payer-provider) covering a quarter of the Israeli population. The database contains longitudinal data on a stable population of ~2.5 million people. Data collected include demographic details, physician visits, laboratory results from a single central laboratory, imaging results, and prescription and drug purchase data. The database includes several automatically formulated registries, including a cardiovascular registry [13]. The MHS ethics committee reviewed the study design and approved it. 

### 2.2. Study Population

The study population included adult patients > 21 years of age to whom HU was dispensed for at least three consecutive months between the years 2000 and 2015. The time of first HU purchase was defined as the index date. Eligible patients had either a recorded diagnosis of PV (ICD-9 Code 238.4) at any time from 2 years before the index date and through treatment, or blood counts indicative of PV (i.e., hematocrit > 45%, platelets > 400 × 10^9^/L, and white blood cells > 10 × 10^9^/L) for 6 months before or after the index date. Study outcomes were documented from the index date until 31 December 2018, death, or leaving MHS. This retrospective, non-interventional study was reviewed and approved by the MHS Institutional Review Committee and all methods were performed in accordance with the relevant guidelines and regulations. The need for informed consent was waived by the MHS Institutional Review Committee.

### 2.3. Patient Groups

All patients in the study started as “HU-treated” patients. Thereafter, they were categorized into three groups. The first group included patients who were resistant to HU based on standard European LeukemiaNet (ELN) criteria [14]. HU-resistant patients had to have been prescribed HU at a dose of 2 g/day for at least 3 consecutive months (and continue without dose reduction), and their hematocrit to have remained above 45% in at least 80% of tests in the first year after the day of first purchase, OR their blood counts to have indicated platelets > 400 × 10^9^/L AND white blood cells (WBC) > 10 × 10^9^/L in at least 80% of tests in the first year after the first purchase. As phlebotomies were not consistently recorded in the Electronic Medical Records (EMRs), resistance was not based on the need for phlebotomies. 

The second group included patients who were intolerant to HU. For the purposes of this study, intolerance to HU was determined as meeting one or more of the following criteria: (i) ELN-based criteria for hematologic toxicity [9], i.e., blood counts performed at least 3 months after the first purchase of HU and during treatment indicating either neutrophil count < 1 × 10^9^/L or platelets < 100 × 10^9^/L or hemoglobin < 10 g/dL; (ii) ceased purchase of HU or dose reduction from 2 g/day; (iii) prescription of busulfan, IFN-α, or ruxolitinib. Additionally, all patients in the intolerance group must not have met the above-mentioned resistance criteria. Non-hematologic toxicity intolerance, such as the development of leg ulcers, was not captured in our analysis since this is not consistently recorded in the EMRs. 

The third group comprised patients on HU who did not meet the criteria for resistance or intolerance and remained on continuous HU treatment (“stable” group).

### 2.4. Demographic and Outcome Variables

The following were collected from EMRs: demographic data, physician visits, laboratory results, imaging results, drug purchase data, and events captured in the cardiovascular registry, including myocardial infarction (MI), non-MI ischemic heart disease (IHD), peripheral vascular disease (PVD), and stroke/transient ischemic attack (TIA) [13]. Additional data regarding additional outcomes were collected: venous thrombotic events, myelofibrosis, AML, mental health events (defined as at least two visits to a psychiatrist or psychologist, or two or more dispensed psychiatric drugs [antipsychotics, anxiolytics, benzodiazepine derivatives or antidepressants]), overall patient treatment costs (data in the EMRs were available for this outcome only from 2010), and mortality.

### 2.5. Outcome Comparison

**Figure 1:**
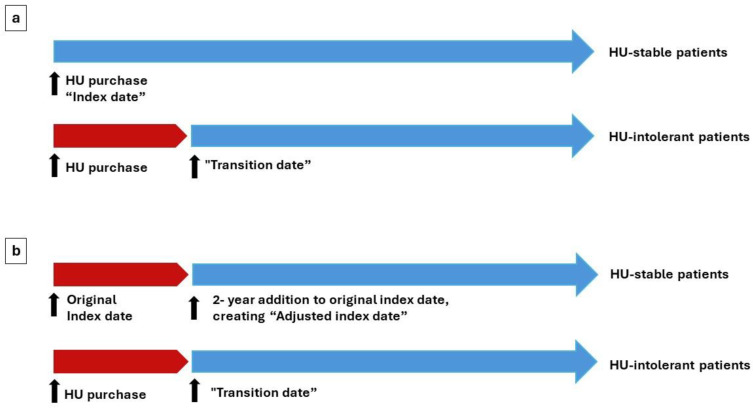
Definition of adjusted index date for the HU-stable group. (**a**) Timepoint of index date for HU-stable patients and timepoint of transition date for HU-intolerant patients. (**b**) Timepoint of adjusted index date for HU-stable patients.

For outcome comparison, a transition date was defined for HU-intolerant patients as the earliest date of cytopenia or stopping HU treatment or receiving second-line treatment (see patient groups, Table 1). To compensate for the time passing from index date to transition date in intolerant patients and to allow a meaningful comparison of HU-stable vs. HU-intolerant patients regarding outcomes that occurred during follow-up, the median time to transition date (~2 years) was added to the index date of stable patients (i.e., stable patients had a new adjusted index date), while patients with shorter follow-up time than the average time to transition (2 years) were not included in the outcome analysis. Only patients transitioning up to 5 years from the index date were included in the outcome analyses, to reduce variability (see illustration in Figure 1). The HU-resistant group was not included in the outcome analysis as only three patients met the resistance criteria (see Section 3). 

### 2.6. Statistical Analyses

Descriptive statistics are presented as number and percent for categorical variables, and as mean ± standard deviation (SD) for continuous variables. Outcomes are reported as number per patient-years, and comparisons of proportions and means across groups were performed using chi-square and Student’s *t*-tests, respectively. Kaplan–Meier survival curves were computed for time to the event and the log-rank test was used to assess between-group differences. 

A multivariate Cox proportional hazards model was used to identify the risk factors for outcomes.

All tests were two-tailed and a *p*-value of 5% or less was considered statistically significant. All analyses were conducted using IBM-SPSS version 25 (IBM-SPSS Statistics for Windows, Version 25, Armonk, NY, USA).

## 3. Results

### 3.1. Study Population

**Figure 2:**
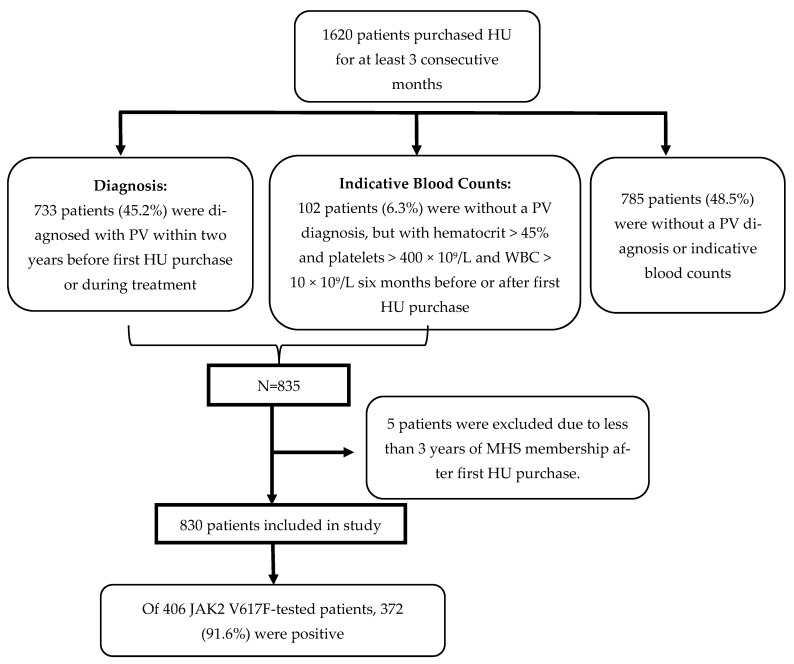
Patient disposition.

A total of 1620 patients who purchased HU for at least three consecutive months were identified, of whom 785 did not have a PV diagnosis or PV-indicating blood counts. Of the remaining 835 patients, 733 (88%) had a diagnosis of PV and an additional 102 (12%) patients had blood counts indicative of PV (with no documented PV diagnosis). Five patients (0.6%) were excluded due to less than 3-year continuous membership in MHS after the index date. Thus, 830 patients remained in the study (Figure 2). Among 406 patients who underwent JAK2 V617F mutational analysis (48.9% of total study patients), 372 (91.6%) were positive (JAK2 V617F testing began in 2007). The annual incidence of PV patients requiring HU treatment during the study period was 2.37 to 5.94 per 100,000 patients. 

### 3.2. Study Groups

Of the 830 study patients, 3 (0.4%) were resistant to HU treatment (HU-resistant group), 318 (38.3%) were intolerant to HU treatment (HU-intolerant group), and 509 (61.3%) were stable on HU treatment (stable-HU group) (Table 1).

Of the 318 HU-intolerant patients, 144 (45.3%) patients developed cytopenia, 52 (16.3%) patients switched to an alternative PV treatment, and 122 (38.3%) patients stopped HU treatment for unknown reasons, and there was no record to indicate that they received any subsequent PV treatment (Table 1).

Of the HU-intolerant patients, 173 (54.4%) transitioned within 5 years from the index date and were compared for outcomes with the HU-stable patients. After compensating for the time interval between the index date and transition date (by adding the median transition time of 2 years to the index date of the HU-stable patients; see Section 2, Figure 1), a total of 487 (95.7%) HU-stable patients with sufficient follow-up time were included. The median follow-up period was 4.9 years for the HU-intolerant group and 5.5 years for the HU-stable group.

### 3.3. Patient Characteristics at Baseline

HU-stable and HU-intolerant patients had similar demographic characteristics at index date: respectively, 57.2% and 54.7% were men and their average age (±SD) was 66.6 (±12.4) years and 65.5 (±13.8) years. No significant differences were observed between the two groups regarding socioeconomic status, hematocrit, hemoglobin, WBCs, lymphocytes, neutrophils, or RBC count; or regarding phlebotomy before HU, history of MI, cerebrovascular accident (CVA), TIA, PVD, or congestive heart failure (CHF) (Table 2). 

A significant difference was noted in red cell distribution width (RDW) and platelet count. The mean RDW was 17.6% and 18.4% (*p* = 0.001), and the mean platelet counts were 495.5 (±226.3) and 430.9 (±258.4) 10^3^/µL (*p* = 0.001) in the stable and intolerant groups, respectively (Table 2).

### 3.4. Patient Characteristics at Baseline after Time Adjustment

For outcome comparison, we present baseline characteristics for patients who were included in the study analysis after time adjustment. The number of non-MI IHD events was significantly higher in the HU-intolerant patients vs. the HU-stable patients (22 [12.7%] vs. 28 [5.7%], respectively; *p* = 0.003). None of the other baseline characteristics differed between the two patient groups (Table 3).

### 3.5. Clinical Outcomes

The results of clinical events are presented in Table 4.

### 3.6. Arterial Cardiovascular Events

No differences were seen in cardiovascular events: the MI event rate was 0.63 and 0.57 per 100 person-years (PY) among HU-intolerant and HU-stable patients, respectively (*p* = 0.836); the non-MI IHD event rate was 1.42 and 0.92 per 100 PY among HU-intolerant and HU-stable patients, respectively (*p* = 0.185); the CVA or TIA event rate was 1.31 and 1.40 per 100 PY among HU-intolerant and HU-stable patients, respectively (*p* = 0.841); and the PVD event rate per 100 PY was 1.42 vs. 0.92 among HU-intolerant and HU-stable patients, respectively (*p* = 0.098).

### 3.7. Venous Thrombosis (Deep Vein, Pulmonary Embolism, and Intra-Abdominal) Events

**Figure 3:**
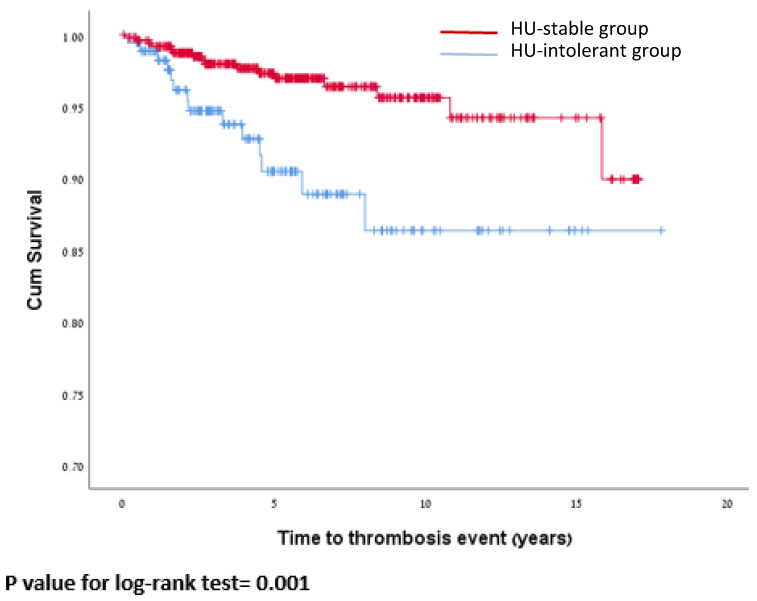
Kaplan–Meier curve for time to venous thrombosis event by group.

The venous thrombosis rate was 1.58 vs. 0.47 per 100 PY for HU-intolerant and HU-stable patients, respectively (*p* < 0.001) (Figure 3). 

### 3.8. Progression to Myelofibrosis or AML

The rate of progression to MF was 6 per 100 PY in the HU-intolerant group vs. 0.9 per 100 PY in the HU-stable group (*p* < 0.001), and progression to AML occurred in 1.16 per 100 PY in the HU-intolerant group vs. 0.2 per 100 PY in the HU-stable group (*p* < 0.001).

### 3.9. Phlebotomies during Follow-up Period

During the follow-up period among all patients, 148 (30.4%) in the HU-stable group and 54 patients (31.2%) in the HU-intolerant group underwent phlebotomy. The mean number of phlebotomies among HU-intolerant patients was significantly higher than in the HU-stable group (9.8 ± 9.5 vs. 6.9 ± 8.0, respectively; *p* = 0.049). 

### 3.10. Hospitalizations

Total hospitalization days per 1 PY were 5.3 among HU-intolerant patients and 1.9 among HU-stable patients (*p* = 0.004). The mean numbers of hospitalizations for the HU-intolerant and HU-stable groups were 6.5 ± 5.4 vs. 3.8 ± 3.5 (*p* < 0.001), respectively (Table 4).

### 3.11. Mortality

**Figure 4:**
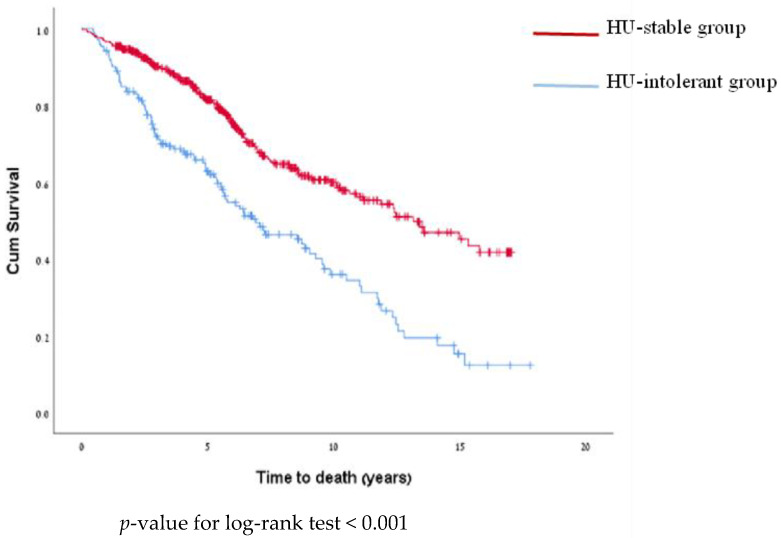
Kaplan–Meier curve for time to death by group.

Death occurred in 58% of HU-intolerant patients vs. 30% of HU-stable patients. The mortality rate was significantly different between the groups: 10.3 per 100 PY in the HU-intolerant group vs. 4.8 per 100 PY in the HU-stable group (*p* < 0.001) (Table 4). The time to death was significantly different between the groups (log-rank test, *p* < 0.001) (Figure 4). 

### 3.12. Mental Health-Related Outcomes

A total of 133 HU-intolerant patients (76.9%) vs. 320 HU-stable patients (65.7%) (*p* = 0.007) had at least two purchases of psychiatric medications or two visits to a psychiatrist/psychologist (Table 4). 

### 3.13. Economic Outcomes

The limitation of expenditure data availability resulted in a total of 82 HU-intolerant patients and 280 HU-stable patients suitable for this analysis. The total healthcare cost over one year from the adjusted index (HU-stable patients) or transition date (HU-intolerant patients) was measured as the total cost ratio. This was defined as the total healthcare cost of HU-intolerant patients divided by the total healthcare cost of HU-stable patients, and resulted in a total cost ratio of 2.65. When the total cost ratio was allocated into four categories of physician visit costs, hospitalization costs, laboratory test costs, and the cost of medications, the highest cost ratio was 3.61 in the hospitalization category (Table 5). 

## 4. Discussion

In this study we show that in routine clinical practice, HU intolerance substantially increases the risk of adverse outcomes in PV patients. These include thrombotic events, progression to MF and AML, the mean number of phlebotomies, hospitalizations, mental health-related events, and mortality. Furthermore, overall financial costs are greater in this patient group.

The prevalence of HU resistance or intolerance in PV patients has been reported to be ~11–24% [5,7,8,9]. In our study, however, we found a higher prevalence of intolerance—38.3%. The ELN guidelines require receiving 2 g/day of HU to define HU-resistance [14]. In this study, we identified very few patients receiving HU at this dose, which limited our ability to study this important group of patients. The reason for this finding is not fully addressed by our study, although it may suggest that many patients reach clinical intolerance before reaching the maximal guideline-recommended dose of HU, demonstrating what could be regarded as “relative resistance” at the patient’s maximal tolerated dose. Our definition of HU intolerance differed somewhat from that of the ELN because we were unable to capture patients with leg ulcers or other HU-related non-hematological toxicities in MHS data. Of note, some patients were defined as intolerant after several months of ceasing HU purchase. We cannot rule out that some of these patients restarted treatment with HU later on.

In our cohort, only 16.3% of the HU-intolerant patients switched to substitute PV treatments (busulfan, IFN-α, or ruxolitinib), while the remainder either continued HU or stopped HU treatment without receiving any substitute treatment. This reflects the limited treatment options that were available to PV patients intolerant of HU during the study period. An additional “follow up” analysis to compare the outcomes of the patients who stopped treatment following HU intolerance with those who switched to a subsequent line of therapy and to those who remained on HU treatment is warranted.

In addition, to better understand the differential impact of HU intolerance based on baseline risk categories, future analyses should stratify patients into low-risk and high-risk groups (e.g., aged > 60 years and/or with a history of thrombosis). This stratification was beyond the scope of the current analysis but could provide additional insights into the management and outcomes of HU-intolerant PV patients. 

### 4.1. Patient Characteristics

While most baseline parameters of stable and intolerant patients were similar, two exceptions are notable—platelet count and RDW. Platelet count was significantly lower in patients who subsequently developed HU intolerance, while RDW was higher in these patients (Table 2). Interestingly, the PV-AIM study (based on the Optum database, utilizing machine-learning technology) identified RDW > 15% as a predictor of HU treatment failure within 3 months in phlebotomy-dependent patients [15], and RDW > 17% as a predictor of HU resistance within 6–9 months of starting HU [16]. Our findings, if validated, may contribute to the development of models to predict HU intolerance.

### 4.2. Clinical Outcomes 

We observed a six-fold higher risk of MF and a five-fold higher risk of AML in HU-intolerant patients. This was comparable to the results in the study by Alvarez-Larran et al. [5], in which HU intolerance or resistance was associated with a 6.8-fold increased risk of transformation to MF or AML. We did not find any significant difference in arterial thrombotic complications, although this may be because of the relatively short follow-up time of five years. However, we found that the venous thrombosis event rate was significantly higher in HU-intolerant patients, suggesting an increased risk of thrombosis in HU-intolerant patients.

Interestingly, in the PV-AIM study, the incidence of thromboembolic events was 20% vs. 15% (restrictive event definition) and 50% vs. 35% (extensive event definition), respectively, in PV patients who continued HU treatment compared to those who switched to ruxolitinib [15]. 

In our study, more HU-intolerant patients than HU-stable patients were hospitalized, and hospitalizations were prolonged among these patients. 

### 4.3. Treatment Costs

The total healthcare cost of HU-intolerant patients was considerably higher than the total healthcare cost of HU-stable patients, most of which was attributable to hospitalization and medication. Previously, Parasuraman et al. reported higher healthcare costs in HU-treated PV patients with the occurrence of thromboembolic events [9], and our study may reconfirm these findings.

### 4.4. Limitations

Our study has important limitations. It is an observational, retrospective cohort analysis of real-world data. Therefore, major components relevant to the clinical course of PV treatment may be underreported or missing, such as leg ulcers and other cutaneous toxicities, phlebotomy frequency, and other potential components of comprehensive documentation. These limitations prevented the implementation of the ELN guideline criteria for determining HU-resistance/intolerance in their full form, thus impeding the direct comparison of our findings with those of prospective observational studies. 

In our study, we identified cytopenia occurring at least 3 months after starting HU. This may reflect a transient situation in some patients until a stable dose of HU was achieved, and thus cytopenia may have resolved with longer follow-up. 

Our study also required a methodological solution to address the fact that HU-intolerant patients had previously been HU-stable by definition, and that stable patients might have developed resistance/intolerance after the study period. We therefore added a 2-year mid-time point to the index date of the HU-stable patients, to enable a meaningful comparison to the HU-intolerant patients, and included in the outcome analysis only patients transitioning up to 5 years from index date, to reduce variability. While adding the mid-time point to the index date of HU-stable patients is in line with previously published studies, we did not conduct propensity score matching [17].

Finally, we did not have information regarding the JAK2 mutational status of some of the patients included, as they were diagnosed and treated prior to the availability of the JAK2 V617F mutation test. Among those tested for the JAK2 617F mutation, 91.6% were positive. This is a little lower than the WHO guideline-referenced percentage of 95% [18], but higher than that reported in other published studies [19]. It is possible that some of the patients with PV diagnosis in their MHS EMR who did not have a documented JAK2 mutation test status in their MHS EMR were actually tested for JAK2 at a hospital and found positive, as part of their overall diagnosis procedure, e.g., prior to the availability of this test in MHS. However, while our findings are in line with previously published data on the adverse outcomes of HU-intolerant PV patients, further studies may be required to fully validate them. 

## 5. Conclusions

In conclusion, this study suggests that in PV, HU intolerance is a risk factor for thromboembolic events, transformation to MF and to AML, and mortality. It also represents a significant economic burden. Thus, surveillance for HU intolerance through the regular monitoring of blood counts and clinical signs is crucial. Signs of HU intolerance to watch out for may include hematologic toxicity such as cytopenia (i.e.,: neutrophil count < 1 × 10^9^/L), platelet count < 100 × 10^9^/L, hemoglobin < 10 g/dL); non-hematologic toxicity such as the development of leg ulcers, gastrointestinal symptoms, and mucocutaneous toxicities; and a lack of efficacy that may present, for example, as a persistent elevation of hematocrit or platelet count despite tolerable HU dosing. Predisposing factors such as RDW or platelet count at baseline should also be monitored and evaluated. Recent data show that IFN-α may lead to better overall survival compared to HU [20], and that ruxolitinib is associated with improved event-free survival and other outcomes compared to best available therapy in HU-resistant/intolerant patients [12,21]. The early and close monitoring of HU-treated patients for signs of intolerance may allow the timely identification of patients in whom the initiation of these drugs may be appropriate and consequently lead to better patient outcomes as well as reduced costs for the healthcare system.

## Figures and Tables

**Table 1:** Study population by study group.

Patient Group	Number of Patients (*n*)	Percentage (%)
HU-resistant patients	3	0.4
HU-intolerant patients	318	38.3
Cytopenia * patients	144	17.3
Alternative PV treatment ** patients	52	6.3
Patients stopping HU	122	14.7
HU-stable patients	509	61.3
**Total patients**	830	100

HU: Hydroxyurea; PV: Polycythemia vera. * cytopenia referred to lab results of either neutrophil count < 1 × 10^9^/L or platelets < 100 × 10^9^/L or hemoglobin < 10 g/dL at least 3 months after the first purchase of HU and during treatment. ** Alternative PV treatment referred to the following: busulfan, IFN-α, or ruxolitinib.

**Table 2:** Patient characteristics at baseline.

	HU-Intolerant Patients (*n* = 318)	^‡^ HU-Stable Patients (*n* = 509)	*p*-Value
Demographics			
Male, *n* (%)	174 (54.7)	291 (57.2)	0.489
Age (years) at index date, mean (SD)	65.5 (13.8)	66.6 (12.4)	0.248
Lab values at first HU ^†^, mean (SD)			
Hematocrit (%)	47.6 (5.2)	47.8 (4.8)	0.615
Hemoglobin (g/dL)	15.2 (1.8)	15.4 (1.7)	0.278
WBCs (×1000/µL)	10.2 (4.9)	11.0 (10.0)	0.248
Lymphocytes (%)	21.5 (9.5)	21.7 (8.5)	0.716
Neutrophils (%)	7.1 (4.1)	7.5 (7.2)	0.439
RBCs (×1000/µL)	5.9 (1.0)	5.8 (1.0)	0.195
RDW(×1000/µL)	18.4 (3.2)	17.6 (3.0)	0.001
Platelets (×1000/µL)	430.9 (258.4)	495.5 (226.3)	0.001
Cardiovascular disease at first HU			
MI, *n* (%)	18 (5.7)	35 (6.9)	0.487
CVA, *n* (%)	10 (3.1)	21 (4.1)	0.47
TIA, *n* (%)	9 (2.8)	18 (3.5)	0.578
PVD, *n* (%)	18 (5.7)	23 (4.5)	0.462
CHF, *n* (%)	7 (2.2)	13 (2.6)	0.748
Phlebotomies before HU, *n* (%)	71 (41)	176 (36.1)	0.253

^‡^ HU-stable patients: *n* = 446; ^†^ Laboratory values for HU-intolerant patients: *n* = 239. CHF: Congestive heart failure; CVA: Cerebrovascular accident; HU: Hydroxyurea; MI: Myocardial infarction; PV: Polycythemia vera; PVD: Peripheral vascular disease; RBCs: Red blood cells; RDW: Red blood cell distribution width; SD: Standard deviation; TIA: Transient ischemic attack; WBCs: White blood cells.

**Table 3:** Baseline characteristics at time of index date adjustment (for patients who were included in the study analysis).

	HU-Intolerant Patients at Transition Date (*n* = 173)	HU-Stable Patients at Adjusted Index Date (*n* = 487)	*p*-Value
Demographics			
Male, *n* (%)	98 (56.6)	277 (56.9)	0.958
Age (years) at index date, mean (SD)	69.5 (13.7)	68.0 (12.2)	0.17
Cardiovascular disease			
MI, *n* (%)	12 (6.9)	32 (6.6)	0.868
IHD non-MI, *n* (%)	22 (12.7)	28 (5.7)	0.003
PVD, *n* (%)	12 (6.9)	27 (5.5)	0.505
CVA/TIA, *n* (%)	20 (11.6)	38 (7.8)	0.134
Thrombosis, *n* (%)	16 (9.2)	34 (7)	0.333

CVA: Cerebrovascular accident; HU: Hydroxyurea; IHD: Ischemic heart disease; MI: Myocardial infarction; PVD: Peripheral vascular disease; SD: Standard deviation; TIA: Transient ischemic attack.

**Table 4:** Summary of clinical outcomes.

	HU-Intolerant Group	HU-Stable Group	*p*-Value
Cardiovascular disease			
Any MI event, *n* (%)	6 (3.5)	17 (3.5)	0.989
Rate per 100 PY	0.63	0.57	0.836
Any IHD non-MI event, *n* (%)	13 (7.5)	27 (5.5)	0.351
Rate per 100 PY	1.42	0.92	0.185
Any PVD event, *n* (%)	13 (7.5)	24 (4.9)	0.204
Rate per 100 PY	1.41	0.81	0.098
Any CVA/TIA event, *n* (%)	12 (6.9)	41 (8.4)	0.538
Rate per 100 PY	1.31	1.40	0.841
Thrombosis			
Any thrombosis event, *n* (%)	14 (8.1)	14 (2.9)	0.003
Rate per 100 PY	1.58	0.47	<0.001
Phlebotomy			
Any phlebotomy, *n* (%)	54 (31.2)	148 (30.4)	0.840
No. of phlebotomies, mean (SD) Median	9.8 (9.5)6	6.9 (8.0)3	0.049
Myelofibrosis			
Myelofibrosis after index, *n* (%)	48 (27.7)	26 (5.3)	<0.001
Rate per 100 PY	6.0	0.9	<0.001
AML			
AML after index, *n* (%)	11 (6.4)	6 (1.2)	<0.001
Rate per 100 PY	1.16	0.2	<0.001
Mortality			
Death, *n* (%)	100 (57.8)	147 (30.2)	<0.001
Rate per 100 PY	10.3	4.8	<0.001
Hospitalization			
Any hospitalization, *n* (%)	145 (83.8)	335 (68.8)	<0.001
First hospitalization: rate per 100 PY	39.7	20.7	<0.001
Total hospitalization days: no. per 1 PY	5.27	1.93	0.004
Only for hospitalized patients	*n* = 145	*n* = 335	
No. of hospitalizations, mean (SD)	6.5 (5.4)	3.8 (3.5)	<0.001
Total hospitalization days, mean (SD)	35.1 (33.3)	17.6 (24.6)	<0.001
Time to first hospitalization			
Mean time, 95% CI (years)	2.75 (2.07, 3.42)	4.83 (4.31, 5.35)	<0.001
Median time, 95% CI (years)	1.05	3.25	
Mental health-related outcomes			
Any visit/medication, *n* (%) ^†^	133 (76.9)	320 (65.7)	0.007
Any visit/medication per 100 PY	13.8	10.5	0.005

^†^ At least two visits or two purchases. CHF: Congestive heart failure; CVA: Cerebrovascular accident; IHD: Ischemic heart disease; MI: myocardial infarction; PV: Polycythemia vera; PVD: Peripheral vascular disease; PY: Person years; SD: Standard deviation; TIA: Transient ischemic attack.

**Table 5:** Healthcare cost ratio in study groups.

	Cost Ratio: HU-Intolerant/ HU-Stable ^†^
Physician Visits	1.07
Hospital Cost	3.61
Lab Test Cost	2.15
Medications	3.28
Total Costs	2.65

HU: Hydroxyurea. ^†^ After one year of adjusted index date/transition date; HU intolerant group *n* = 82; HU-stable group *n* = 280.

## Data Availability

The datasets generated during and/or analyzed during the current study are not publicly available due to ownership by MHS but may be available via the corresponding author upon reasonable request.

## Notes

**Key findings of this study were presented virtually as a poster presentation at the 62nd annual meeting and exposition of the American Society of Hematology (ASH), December 2020. Blood (2020) 136 (Supplement 1): 23–24. https://doi.org/10.1182/blood-2020-141821.**
